# ROCK-1 mediates diabetes-induced retinal pigment epithelial and endothelial cell blebbing: Contribution to diabetic retinopathy

**DOI:** 10.1038/s41598-017-07329-y

**Published:** 2017-08-18

**Authors:** Pierre-Raphaël Rothschild, Sawsen Salah, Marianne Berdugo, Emmanuelle Gélizé, Kimberley Delaunay, Marie-Christine Naud, Christophe Klein, Alexandre Moulin, Michèle Savoldelli, Ciara Bergin, Jean-Claude Jeanny, Laurent Jonet, Yvan Arsenijevic, Francine Behar-Cohen, Patricia Crisanti

**Affiliations:** 1 Inserm UMR_S 1138, Team 17: From physiopathology of retinal diseases to clinical advances, Centre de Recherche des Cordeliers, Paris, France; 2 Sorbonne University, University of Pierre et Marie Curie, UMR_S 1138, Centre de Recherche des Cordeliers, Paris, France; 3 Paris Descartes University, Sorbonne Paris Cité, UMR_S 1138, Centre de Recherche des Cordeliers, Paris, France; 4 Department of Ophthalmology of University of Lausanne 1000 Lausanne, Jules Gonin Hospital, Lausanne, Switzerland; 5 Department of Ophthalmology, Assistance Publique-Hopitaux de Paris, Hôtel-Dieu de Paris Hospital, 75004 Paris, France; 6 INSERM U1138 Team 17, Le Centre de Recherches des Cordeliers (CRC), 75006 Paris, France; 7 University of Lausanne, Lausanne, Switzerland

## Abstract

In diabetic retinopathy, the exact mechanisms leading to retinal capillary closure and to retinal barriers breakdown remain imperfectly understood. Rho-associated kinase (ROCK), an effector of the small GTPase Rho, involved in cytoskeleton dynamic regulation and cell polarity is activated by hyperglycemia. In one year-old Goto Kakizaki (GK) type 2 diabetic rats retina, ROCK-1 activation was assessed by its cellular distribution and by phosphorylation of its substrates, MYPT1 and MLC. In both GK rat and in human type 2 diabetic retinas, ROCK-1 is activated and associated with non-apoptotic membrane blebbing in retinal vessels and in retinal pigment epithelium (RPE) that respectively form the inner and the outer barriers. Activation of ROCK-1 induces focal vascular constrictions, endoluminal blebbing and subsequent retinal hypoxia. In RPE cells, actin cytoskeleton remodeling and membrane blebs in RPE cells contributes to outer barrier breakdown. Intraocular injection of fasudil, significantly reduces both retinal hypoxia and RPE barrier breakdown. Diabetes-induced cell blebbing may contribute to ischemic maculopathy and represent an intervention target.

## Introduction

The retina, part of the central nervous system is isolated from the rest of the organism by two blood-retinal barriers. Tight-junctions of endothelial cells in retinal capillaries form the inner blood-retinal barrier and tight-junctions in the retinal pigment epithelium (RPE) form the outer-retinal barrier. Retinal barriers tightly control exchanges between the retina and the systemic circulation and, in partnership with retinal glial Müller cells, maintain the hydro-ionic retinal homeostasis. Diabetic retinopathy (DR) is a growing cause of blindness worldwide^1^, the retina being a major site of diabetes-induced microangiopathy. The grading and severity of diabetic retinopathy relies on microvascular lesions, but novel imaging and exploration methods have shown that outer retinal barrier breakdown and neuro-degeneration precede ophthalmoscopy changes^2, 3^. More recent vascular imaging using optical coherence tomography-angiography revealed reduction in capillary flow as a surrogate marker of retinopathy severity^4^. Indeed, any reduction in the lumen of retinal capillaries that are less than 10 µm in diameter may induce retinal ischemia^5^.

To date, therapeutic options for DR have included laser photocoagulation of the peripheral ischemic retina to avoid neovascularization and, intraocular injection of anti-edematous drugs (anti-VEGF and corticoids)^6^. If severe complications have been reduced, major causes of visual impairment still remain macular edema and macular ischemia. A better understanding in the molecular mechanisms causing microvascular occlusion and barriers breakdown could further improve therapeutic interventions. Amongst the cellular functions altered by diabetes, we have focused our analysis on cytoskeleton alterations.

Indeed, through cytoskeleton remodeling, the downstream effectors of small Rho GTPases, ROCK-1 and ROCK-2, not only regulate junction proteins, but also contribute to cell polarity, mobility and apoptosis^7^. Indeed, ROCK-1 controls endothelial cell shape modifications and pericytes contractility^8, 9^ and is predominant in epithelial polarized cells^10–12^. Abnormal ROCK-1 activation has been shown in various vascular diseases such as hypertension^13, 14^, coronary and cerebral vasospasm^15, 16^, atherosclerosis^17^, stroke^18^, pulmonary hypertension^19^, and cardiovascular diseases^20^. Rho kinase inhibition showed beneficial effects in diabetes-induced neuropathy^21^, nephropathy^22^, and cardiopathy^23^. In streptozotocin-induced type-1 diabetes, the Rho/Rock pathway activation contributes to leukocytes adhesion and to subsequent endothelial cell death in retinal capillaries^24^. As a consequence of its effects on cell contractility, ROCK may also intervene in the development of pre retinal neovascularization and in tractional retinal detachment^25^. To our knowledge, only one pilot study was published showing that intraocular fasudil, a specific ROCK inhibitor, combined with intravitreal bevacizumab (anti-VEGF) was beneficial for patients with macular edema resistant to anti-VEGF alone^26^.

In the Goto-Kakizaki (GK) type 2 diabetic rat model, activity of the atypical PKC ζ which phosphorylates ROCK^27^ is regulated early in the retina^28^ and interaction of PKC ζ with RhoA is required for LPS-induced blood-brain barrier breakdown^29^ suggesting that Rho-kinases could intervene in retinal barriers alteration in the diabetic retina. But, the early consequences of diabetes on ROCK-1-induced cytoskeleton regulations remain imperfectly understood. In this study, we have evaluated the effects of ROCK-1 activation in the inner and outer retinal barriers components in the GK rat.

## Research Design and Methods

### Human ocular tissues

The use of human subjects adhered to the tenets of the Declaration of Helsinki and was approved by the local Ethics Committee of the Swiss Department of Health on research involving human subjects (CER-VD N°340/15 and CER-VD N°19/15) and patients signed an informed consent. Two fresh retinas were obtained from enucleated eyes of patients with extra ocular or uveal tumors but intact retinas (untreated superior uveal melanoma and conjunctival mucoepidermoid carcinoma). One patient suffered from type2 diabetes for 23 years (female, 67 years-old), had diabetic macular edema and several systemic complications (diabetic nephropathy with end-stage renal failure and dialysis and peripheral neuropathy). The second eye was from a non-diabetic patient (female, 56 years-old). The enucleated eyes were sectioned and the anterior part (including retina up to the equator) was used for classical pathologic examination. The posterior retinas of both eyes were used for immunohistochemistry on cryosections. Due to the enucleation procedure, fresh tissues were available for analysis.

### Animal Model and fasudil treatment

Animal experiments followed the European Community guidelines and were approved by the local Ethical Committees of Paris Descartes University and validated by the French Ministry of Research and were registered (Ce5/2012/085, Ce5/2012/080, and Ce5–2009–034). In accordance with the “3 R” rules, experiments were designed to reduce the number of animals (10 animals per group at the maximum) and the most relevant time points for animals to be sacrificed. Goto-Kakizaki (GK) rats (Taconic Europe, Denmark), a Wistar strain of non-obese, type 2 diabetes, were studied at 12 months of age. Non-fasting blood glucose was measured using Accutrend GC and Accu-check compact equipment (Roche) and HbA1c was measured with A1C NOW + multitest system (Bayer, Germany). A plasma glucose level >250 mg/dL (14 mmol/L) defined the diabetic status. In contrast to control Wistar rats, GK rats develop hyperglycemia at approximately 14 weeks of age (Table 1). Control age-matched Wistar rats (WS) were normoglycemic. Three consecutive intravitreous injections were performed. Either fasudil (at a concentration of 20 μM) or vehicle (PBS) was injected, at 48 hours interval. Animals were killed 48 hours following the last administration. There were 6 groups of 12-months old Wistar and age-matched GK rats, receiving fasudil, PBS or control.

**Table 1:** Characteristics of the study animals.

	Control (*WS*)	Diabetes (*GK*)	*p* value
Number of animals	40	40	
Weight (g)	568 ± 55	383 ± 35	<0,001
Glycemia (mg/dl)	269 ± 15	401 ± 69	<0,001
HbA1c (%, mmol/mol)	4,4 ± 0,2 (24 ± 3)	8,7 ± 1,4 (72 ± 12)	<0,001

Data are mean ± SD;

12-month-old male type 2 diabetic Goto-Kakizaki (GK) and 12-month-old male Wistar (WS) rats.

### Retinal cryosections, retinal flat-mounts, semi-thin and ultrathin sections

Rat eyes were enucleated for cryosections as previously described^30^. For retina flat-mounts the neural retina was separated from the RPE/choroid complex and then processed as previously described^31^. For semi-thin and ultrathin sections, eyes from GK rats (*n* = 8 rats per time point) were fixed 1 hour in 2.5% glutaraldehyde in cacodylate buffer (0.1 mol/L, pH7.4) and then dissected, post-fixed in 1% osmium tetroxide in cacodylate buffer, and dehydrated in a graded series of alcohol before being included in epoxy resin and oriented. Semi-thin sections (1 μm, ultra-microtome Reichert Ultracut E (Leica), were stained with toluidine blue. Ultrathin sections (80 nm) were contrasted by uranyl acetate and lead citrate and imaged with a transmission electron microscope (TEM, JEOL 100 CX II (JEOL) with 80 kV).

### Immunohistochemistry on neural retina or RPE/choroid

Immunohistochemistry was repeated at least 7 times on 7 different animals for each group. Negative controls were obtained by staining procedures that omitted the primary antibody (data not shown). The list of antibodies used in this study is provided in Table 2. Confocal imaging and photographs were taken using the Confocal Zeiss LSM710. For 3D images, projections-Plot from image stacks showing fluorescence intensity information from a composite image of multiple channels of fluorochromes was used. Internally the image was scaled to a square image using nearest neighbor sampling. The surface plot showed the color distribution within a 3D-color-space. The luminance of an image is interpreted as height for the plot.

**Table 2:** List and details of antibodies.

	Species	Reference	Lab provider	dilution
Anti-occludin (polyclonal)	Rabbit	71–1500	Zymed, San Francisco, CA, USA	1/200
Rhodamin phalloidin		R415	Life technologies, Eugene, Oregon, USA	1/300
Anti ROCK-1	Mouse	sc-17794	Santa Cruz Biotechnology, Inc, Santa Cruz, CA, USA	1/200
Anti ROCK-1	Rabbit	sc-5560	Santa Cruz Biotechnology, Inc, Santa Cruz, CA, USA	1/200
Anti ROCK-1P (Thr455/Ser 456) Anti ROCK-2	Rabbit Rabbit	Bs-4630R Ab66320	Bioss antibodies, MA, USA Abcam, Cambridge, UK	1/100 1/100
Anti-Myosin light chain (polyclonal) phospho S20	Rabbit	ab2480	Abcam, Cambridge, UK	1/1000
Lectin from BS –TRITC		L5264–2MG	Sigma Aldrich St. Louis, MO USA	1/200
Anti-actin	Rabbit	A2066	Sigma Aldrich St. Louis, MO USA	1/500(WB)
1/200(IF)				
Anti-Rat VEGF 164	Goat	AF564	R&D Systems, Inc. Minneapolis	1 µg/ml
Cleaved Caspase-3 (Asp175)	Rabbit	#9664	Cell Signaling Technology Danvers, MA	1/100
Alexa Fluor 488 or 594			Invitrogen life technology Carlsbad	1/250
4’, 6-diamino-2-phenylindol (DAPI)			Sigma Aldrich St. Louis, MO USA	1/5000

BS, Bandeiraea simplicifolia.

### ROCK-1 activation

A ROCK activity was measured using Immunoblot Kit (Cell Biolabs, San Diego, CA, USA) Cell Biolabs’ ROCK Activity Immunoblot Kit utilizes recombinant MYPT1 as ROCK substrate. After incubating the substrate with RPE/choroid fresh lysate as samples, the phosphorylated MYPT1 was detected by western blot analysis using an anti-phospho-MYPT1 (Thr696). ROCK inactivates myosin phosphatase through a specific phosphorylation of myosin phosphatase target subunit 1 (MYPT1) at Thr696, which results in an increase in the phosphorylated content of the 20-kDa myosin light chain (MLC20) (Abcam, Cambridge, UK). After incubating the substrate (recombinant MYPT1) with Freshly RPE/choroid samples, the phosphorylated MYPT1 was detected by western blot analysis using an anti-phospho-MYPT1 (Thr696). Inhibition of ROCK activity, was quantified by western blotting.

### Western blotting

RPE/choroid and neural retina were homogenized in a lysis buffer (50 mM Mops, 50 mM Trisbase, 0,1%, SDS 1 mM EDTA PH 7.7) containing a protease inhibitor cocktail (Roche, France). Protein concentration was determined using a Pierce BCA protein assay kit (Thermo Scientific Rockford, USA) (20–40 μg) were subjected to SDS-PAGE on Nupage 4–12% Bis –Tris gel electrophoresis, and electro-blotted onto nitrocellulose membranes (Optitran BA-S 83 GE Healthcare Life Science Whatman). Membranes were incubated with primary antibodies. Then, membranes were incubated with the corresponding peroxidase-conjugated F(ab)2 fragment (Santa Cruz Biotechnology Inc, Santa Cruz, CA, USA) (dilution1:5000) secondary antibodies. Immuno-reactive bands were detected with the ECL Western blotting Detection Reagents Kit (Thermo Scientific Rockford USA). The relative abundance of individual proteins identified was quantified by scanning densitometry. The list of antibodies used for western-blots are provided in Table 2.

### Intravenous injection of FITC-Dextran

To assess blood retinal barrier breakdown, 200 µL of 150 KDa FITC-dextran at 50 mg/mL in PBS (Sigma) was injected intravenously in the tail of rats treated by fasudil or vehicle (n = 5), 2 hours prior rat sacrifice. Leakage of FITC-dextran was evaluated by measuring fluorescence within the retina using ImageJ software as previously described by others^32^ i. Cryo-sections (10 µm) of fasudil and vehicles injected rat retinas were randomly imaged (10 images at 40x magnification per animal) and analyzed.

### Retinal endothelial cell death evaluation « *in vivo* »

Propidium iodide (Molecular Probes) (1 mg/ml) was injected intravenously in the caudal vein. After 12 h, the retinas were then studied under a fluorescence microscope with lectin staining.

### Quantitative assessment of the retinal vessels

#### Vascular surface coverage on retina flat-mounts

The vascular area covered by large and small blood vessels stained with TRITC lectin (Sigma Aldrich St. Louis, MO USA) on flat-mounted retina was quantified using an ImageJ macro in Fiji^33, 34^. Briefly, the local thickness function available with the Fiji plug-in for ImageJ software was used to create a map of the blood vessels, from which diameter of the local structures could be evaluated. This map was threshold to define two binary masks corresponding to the vessels of large diameter (local thickness > threshold) and the vessels of small diameter (local thickness < threshold). The area of those masks was then measured and expressed in terms of the surface area occupied by the small vessels.

#### *In vivo* measurement of vascular diameters

To evaluate the vasodilator effects of fasudil on retinal vessels, confocal fluorescein angiography (FA) was performed before and 2 days after treatment. Briefly, *in vivo* FA were conducted using a HRA-II, spectral domain digital imaging system (Heidelberg Retina Angiograph II, Heidelberg Engineering, Inc., Dossenheim, Germany), which uses a confocal scanning laser ophthalmoscope. Fluorescein (0.3 ml of 10% fluorescein, SERB laboratories, Paris, France) was injected intravenously in the tail vein. After injection, early and late-phase fundus angiograms were taken at 3 different non-overlapping retinal locations. Edges detection was performed using a Canny-Deriche filtering for edge detection on Image J (www.tomgibara.com/computer-vision/canny-edge-detector). Eight measurements of each vessel diameter were performed using the built-in caliper at the same vascular site before and after treatment. Differences in pre- and post-treatment measurements were computed as well as the mean of the differences for each animal. A vasodilation ratio (vessel lumen post-vessel lumen pre)/vessel lumen pre) was used to compare the fasudil group to the vehicle group.

### Evaluation of retinal hypoxia

The effect of fasudil on retinal perfusion and subsequent retinal hypoxia, was evaluated on one-year old GK rats using hypoxyprobe-1 Kit (Hypoxyprobe, Inc. Burlington, MA, USA), classically used to detect hypoxic area. Indeed, pimonidazole incorporates the nitroimidazole moiety serving as biomarker of tissue hypoxia. In tissues with low oxygen pressure, reduced nitroimidazole bound to proteins can be detected^35, 36^.

Three hours before sacrifice, rats treated with fasudil or vehicle (n = 4/group) (3 IVT, 20 µM final, at 48 h intervals, n = 4), received an intraperitoneal pimonidazole hypochloride injection (60 mg/Kg body weight) following the Hypoxyprobe™-1 Kit procedure. Rats were sacrificed and the enucleated eyes were fixed with 4% paraformaldehyde for 1 h, then dissected and the retina were flat-mounted, post-fixed for 1 h, blocked with 3% skim milk for 30 min, incubated with anti-pimonidazole antibody at a dilution of 1:100 in blocking solution overnight together with FITC conjugated Bandeiraea simplicifolia lectin (Sigma Aldrich, Gillingham, UK) at a concentration of 0.1 mg/ml. Retina were then rinsed and incubated a secondary antibody (Alexa Fluor 647-conjugated anti-rabbit polyclonal Ab). Retinas were mounted with fluorescent aqueous mounting medium (Dako Ltd., Ely UK), examined and images acquired with confocal microscope with mosaic and Z-stacks (LSM 510 laser scanning microscope Zeiss, Carl Zeiss, Le Pecq, France). The hypoxic area identified by pimonidazole labeling, were measured using ImageJ software. The total hypoxic area and the total retinal area were measured and the hypoxic area ratio calculated.

### Quantitative assessment of RPE cell layer morphology

RPE cell morphology was assessed on RPE flat- mounts using an ImageJ^32^ macro tool created in Fiji^31^. Briefly, after a first image processing by FFT band pass filtering, images were converted into binary format and masked and then processed to obtain a skeleton image (isolated segments and loops were removed). This image was inverted and the cells segmented and labeled with the “particle analysis” tool in ImageJ, the Regions of Interest (ROI) were delimited using ROI Manager tool. The number of vertices for each cell was measured following detection of triple points (points were three branches are joined together) with the appropriate binary “hit or miss” transformation. For this transformation, the ImageJ plug-in “morphology” was used. By removing those points from the skeleton image, the number of individual edges was counted as well as their length. The number of vertices was then verified and manually corrected for errors. Images with more than 5% automatic alignment errors were eliminated from analysis. Following this, several morphological parameters were then measured for each cell (e.g. area, perimeter, circularity, number of neighbors/vertices/edges of a cell).

### Statistics

For continuous variables, the mean ± SD were provided. Comparisons were performed using the non-parametric, Mann–Whitney test (Prism software version 4.0c; GraphPad Software, San Diego, CA), p-values < 0.05 were considered significant. Multiple mean comparisons were performed using analysis of variance (ANOVA) with repeated measures (R software version 3.2.2).

## Results

### Dual consequence of ROCK-1 activation on diabetic retinal microvessels

#### Involvement of ROCK-1 in vasoconstriction

**Figure 1:**
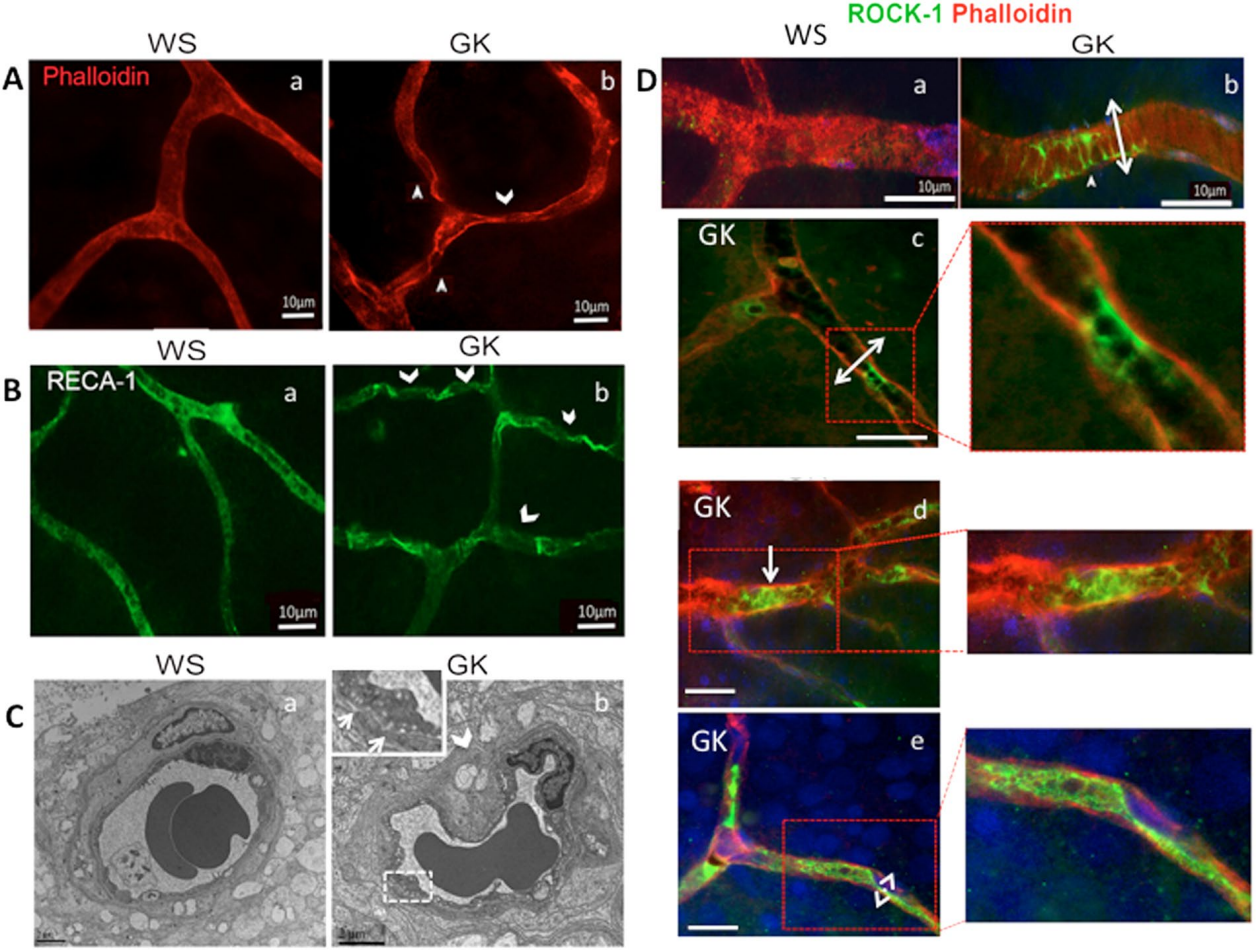
ROCK-1 activation in micro vessels of diabetic retina (**A**). Phalloidin-stained actin on flat-mounted retina vessels of 12 months-old rats. *a*- WS control rat, *b*- GK rat showing focal vasoconstrictions (arrowheads). (**B)** RECA-1 stained endothelial cells on flat-mounted retina vessels of 12 months-old rats. a-WS control rats, *b*- GK rats showing irregular labeling (arrowheads). (**C**) Transmission electron microscopy (TEM) showing micro vascular constriction (arrowhead) and vacuoles in endothelial cells (inset) but preserved nuclei in GK rat retinal vessels as compared to normal WS structure. (**D**) ROCK1-phalloidin double staining (ROCK-1 in green, phalloidin in red and DAPI in blue) *a*–*b*: on flat-mounted retina, ROCK-1 is faintly expressed in WS rat vessels but is located in constricted area where smooth muscle cells are contracted (b, double arrow). *c*–*e*: on GK retina sections, focal ROCK-1 expression is located in the endoluminal side of constricted vessel (c and magnification), in aggregated luminal cells (d and magnification) and at the membrane of endothelial cells where the vascular lumen is diminished (e, double arrow and magnification). Scale bar = 10 µm, for TEM scale bar = 3 µm.

On retinal flat-mounts, phalloidin-stained actin bordered the regular vessels walls and RECA-1 (Rat Endothelial Cell Antigen) labeled continuous endothelial cells of Wistar control rats (WS, n = 8), but showed the focal constrictions of retinal vessels and irregular endothelial cells of diabetic rats GK, n = 8) (Fig. 1A and B, white arrowheads). Constricted capillaries with vacuolized endothelial cells were more obvious on ultra-thin sections of GK rat retina (Fig. 1C). ROCK-1 was expressed externally in mural cells at the level of constricted area (Fig. 1D,b, double arrow) and internally in the inner vessel wall (Fig. 1D,c, double arrow). Aggregated ROCK-1 positive cells filled the lumen of constricted vessels (Fig. 1D,d, white arrow).

#### Involvement of ROCK-1 in endoluminal membrane blebbing

**Figure 2:**
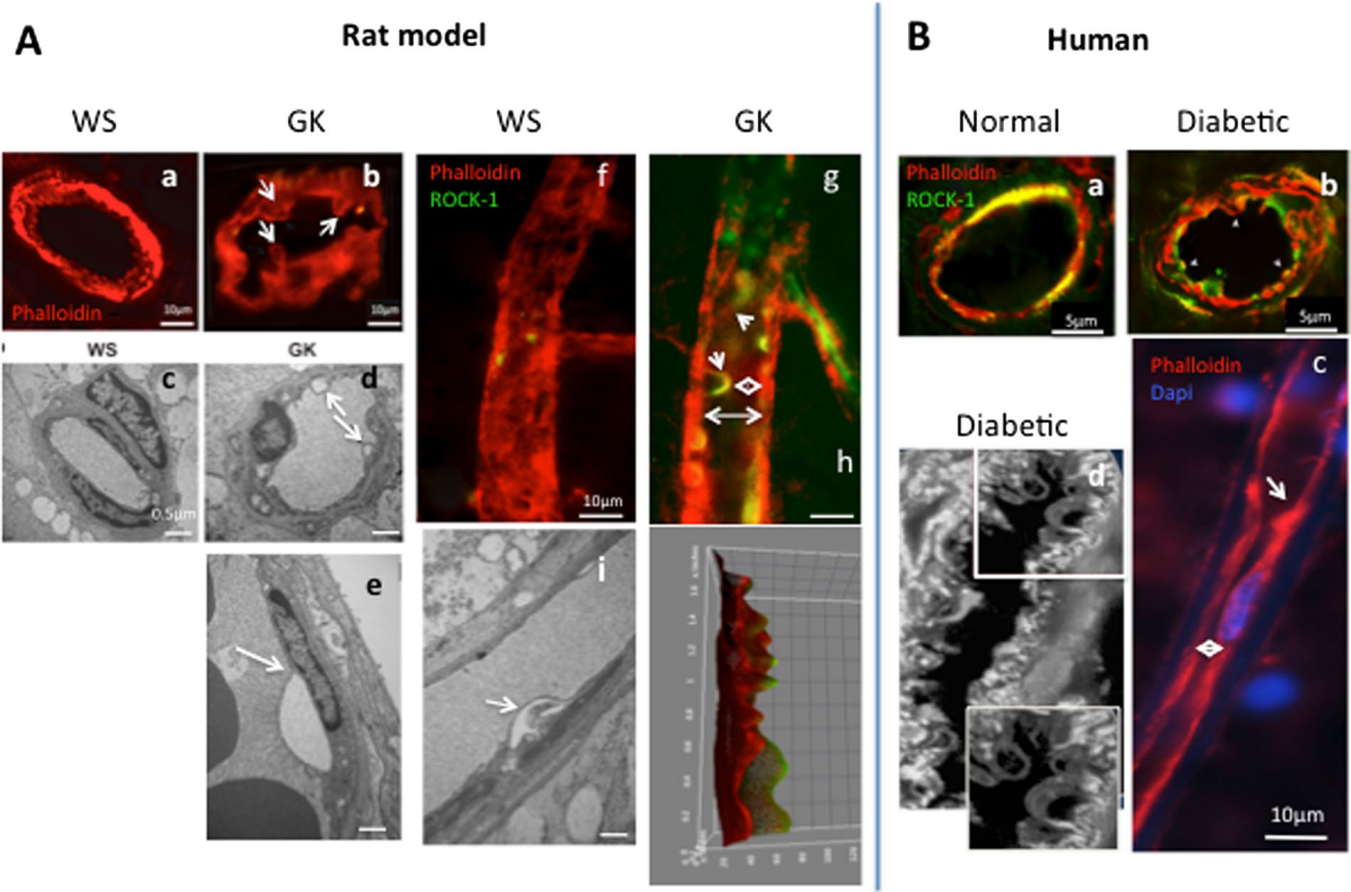
ROCK-1 distribution and blebbing in endothelial cells of diabetic retinal vessels. ROCK1-phalloidin double staining (ROCK-1 in green, phalloidin in red) on: (**A**) Rat retina, (**B)** Human retina. Endothelial cell blebbing is identified by actin staining only in GK rat retinal vessels (**A**,a–b). TEM confirms cytoplasmic protrusions with double layered membrane and normal nuclei in GK rats vessels and not in WS rats (**A**,b–e). In GK rat vessels, ROCK-1 is distributed at the endothelial cell membrane and surrounds the endoluminal blebs (**A**,g arrows) that reduce the lumen (**A**,g, double arrow). 3D representation of ROCK-1 and phalloidin staining clearly show the membrane protrusions co-stained with ROCK-1 and phalloidin (**A**,h). In vessels from a normal human retina, ROCK-1 co-localizes in the cytoplasma of endothelial cells in retinal vessels (**B**,a), but is translocated at the membrane and in blebs in endothelial cells from a diabetic human retina (**B**,b), where intense blebbing can be observed (**B**,c) narrowing the vessel lumen. Scale bar = 10 µm, for TEM scale bar = 0.5 µm.

On retinal sections from GK rats, closer examination of endoluminal protrusions showed endothelial cell membrane blebs (Fig. 2A,b, white arrows), apparently not associated with apoptosis at this stage of diabetes since intravenous propidium iodide, known to stain late stage apoptotic and necrotic cells, did not stain endothelial cells of GK rats. Moreover, activated caspase-3 was not observed in endothelial cells from GK rats (not shown). Membrane blebbing was confirmed on ultra-thin sections, showing double–layered membrane protrusions from endothelial cells and non-fragmented nuclei (Fig. 2A,d and e, white arrows).

ROCK-1 localized in blebs/protrusions of the vascular endothelium (Fig. 2A,f and g, arrowheads) since membrane blebbing results from Rock-1-induced cortical actin contraction^37^. The endoluminal blebs induced narrowing of the vessel lumen (Fig. 2A,g, double arrow). The 3D color distribution better represented the endoluminal bleb protrusion (Fig. 2A,h). Similar observations emerged from human retina immunohistochemistry analysis. In the retinal vessels from human diabetic eye, ROCK-1 appeared localized at the membrane of endothelial cells and filled endoluminal blebs (Fig. 2B,a andb). On magnified images, blebs extended inside the vessel lumen (Fig. 2B,d). Longitudinal section of capillaries showed reduced lumen (Fig. 2B,c, double arrow) and intraluminal protrusion (Fig. 2B,c, arrow).

These observations indicate that ROCK activation contributes to the vessel lumen diameter reduction both through vessels constriction and through non-apoptotic endoluminal blebbing.

### Activation of ROCK-1 in diabetic RPE is associated with cytoskeleton remodeling and outer blood retinal barrier breakdown

**Figure 3:**
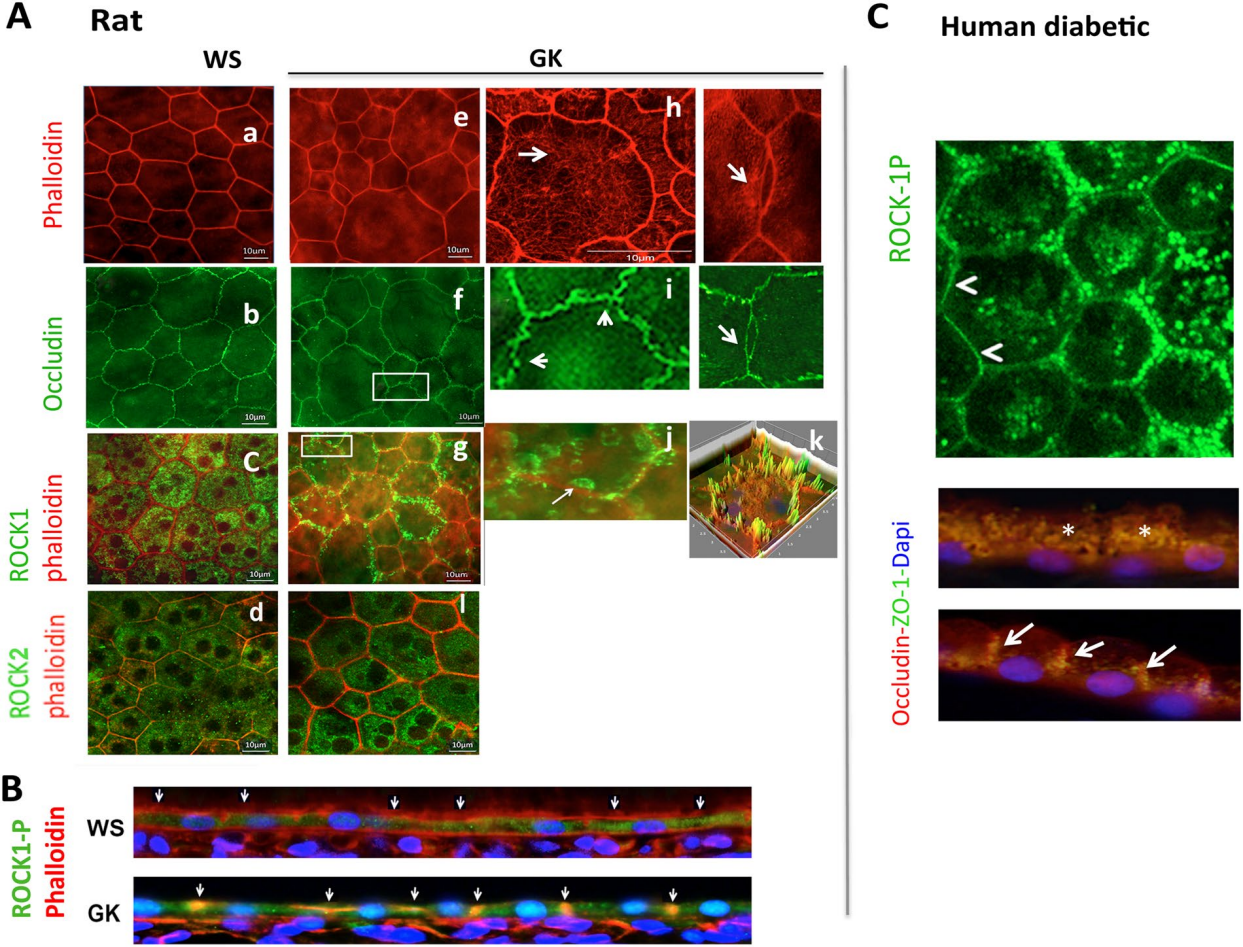
ROCK-1 and ROCK-2 distribution in diabetic retinal pigment epithelial cells (RPE) and ROCK-1 recruitment in membrane blebbing. ROCK1-phalloidin double staining (ROCK-1 in green, phalloidin in red and DAPI in blue) and occludin (green) on: (**A,B)** Rat retina, (**C**): Human retina. In non-diabetic WS rats, phalloidin labelled the regular hexagonal cytoskeleton of RPE cells (**A**,a). In GK rats, cytoskeleton rearrangement with shape and size modifications of RPE resulting in cell constrictions with reduced area and stress fiber formation are observed (**A**e,h). Regular occludin stain the WS RPE (b), but focal occluding disruption and opening are observed in GK RPE (f,i). ROCK-1 is cytoplasmic in WS but translocated at the membrane in GK RPE (c,g,j,k) and ROCK-1 positive blebs are observed in GK RPE (**A**,j). Transversal section of RPE confirms the membrane translocation of ROCK-1 in the diabetic RPE (**A**,k), demonstrating that ROCK-1 is activated in GK RPE. Similar faint membrane and perinuclear distribution of ROCK-2 is observed in WS and GK rats (d,i). (**C**) In human RPE, ROCK-1 is recruited at the membrane (white arrows on flat-mounts and on RPE section). Scale bar = 10 µm.

Phalloidin, which stains F-actin, lined the regular hexagonal shape of RPE cells from control WS rat (Fig. 3A,a) but showed poly-dispersed (constricted or enlarged) RPE cells with focal stress fibers at the level of the subcortical actin in GK rats (Fig. 3A,e and h, white arrows). Whilst occludin regularly stained tight-junctions of non-diabetic WS rat (Fig. 3A,b), focal junction opening were observed in the diabetic GK rat (Fig. 3A,i) white arrows) together with stress fibers. Diabetes induced a striking change in ROCK-1 subcellular distribution, displaced from the cytoplasm to the membrane, co-localizing with F-actin (Fig. 3A,g,j and k) and responsible for the apical cellular constriction. Transversal RPE sections confirmed the membrane localization of ROCK-1P in GK rats and its cytoplasmic localization in the control rat (Fig. 3B). In GK rats, ROCK-1 also stained RPE membrane blebs (Fig. 3A,g,j). Similarly, on human flatmount and transversally sectioned RPE from a diabetic donor, membrane localization of ROCK-1 was observed (Fig. 3C, white arrows). However, in some area, ROCK-1 remains located in the cytoplasma (Fig. 3C, section, white stars), confirming the membrane recruitment of ROCK-1 in the RPE in diabetic conditions in human.

We also analyzed the effect of diabetes ROCK-2 distribution in RPE cells. ROCK-2 faintly stains the cytoplasmic membrane, but mostly concentrated in perinuclear regions in both the GK and the WS control rats (Fig. 3A,d,l). Diabetic conditions do not significantly modify the sub-cellular distribution of ROCK-2.

### Intravitreous injection of fasudil inhibits ROCK over-activation in RPE cells

**Figure 4:**
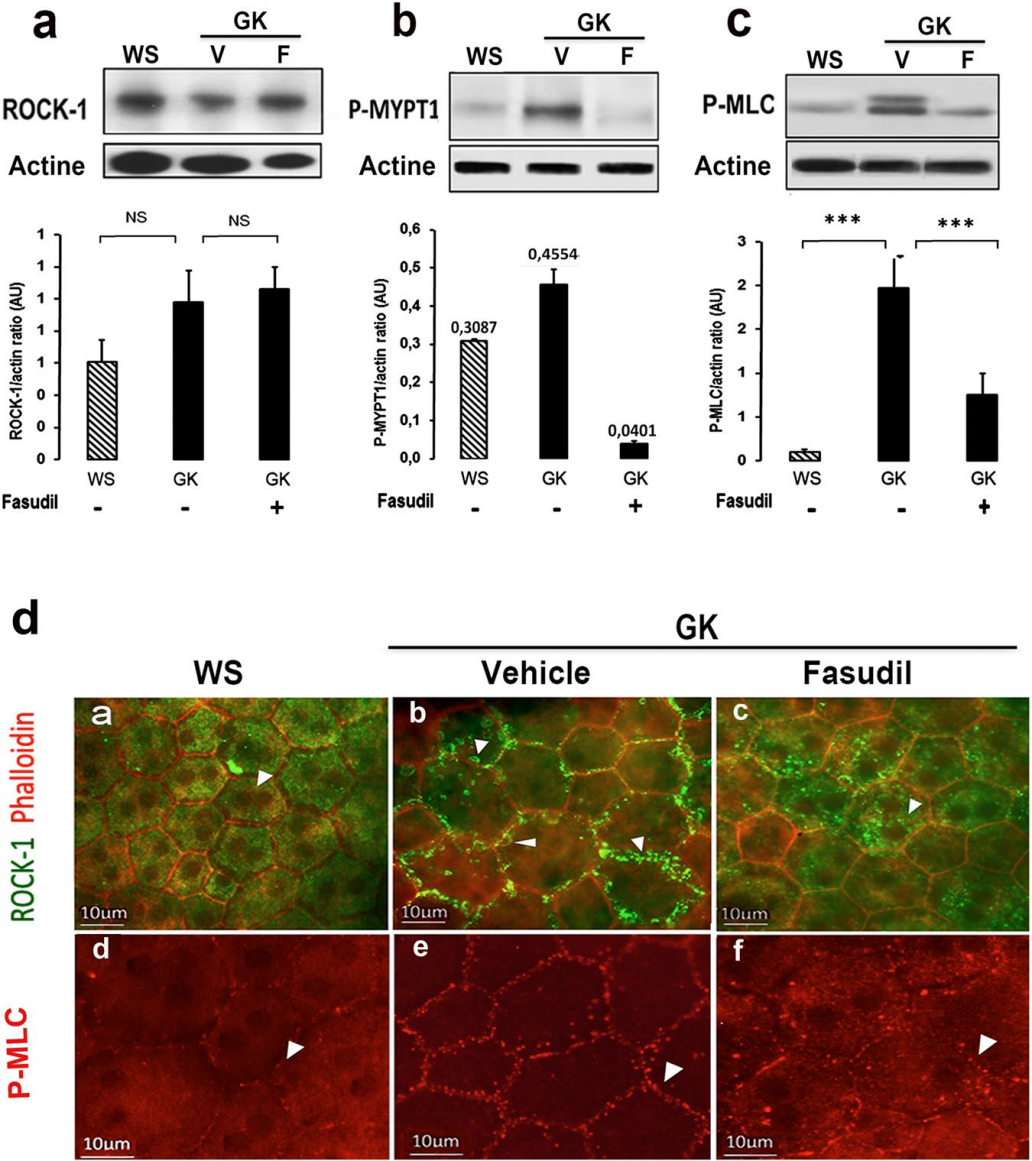
Effect of intravitreal fasudil on ROCK-1 activation state. Western blotting analyses to assess ROCK-1 protein level showed no significant change in any group with Fasudil treatment in GK rats as compared to vehicle treated GK rats (V) or WS rats (n = 6–7 per group) (**a**). An *in vitro* ROCK activity immunoblot kit evaluating recombinant MYPT1 phosphorylation level was performed and showed a decrease in ROCK activity in GK Fasudil treated rats (**b**) (n = 5 per group). We confirmed ROCK-1 activity inhibition by the significant decrease of MLC phosphorylation in GK Fasudil treated rats (**c**) (n = 12 per group); ***p value < 0.001. In GK rats, activation of ROCK-1 coincides its membrane recruitment and associated with P-MLC at the membrane (**D**,b,e). Fasudil restores the cytoplasmic localization of ROCK-1 and MLC (**D**,c,f).

After three consecutive intravitreous injections of fasudil (20µm) or vehicle (PBS) performed at 48 hours interval, animals were killed 48 hours following the last administration. No significant change in ROCK-1 protein levels was observed between control WS, diabetic GK treated with vehicle or treated with fasudil (Fig. 4,a). But a significant over-activation of ROCK-1 was assessed by the increased phosphorylation state of two of its known substrates: MYPT1 and MLC in the diabetic retina as compared to the control (Fig. 4b,c). Interestingly, the membrane recruitment of ROCK-1 was associated with the phosphorylation and membrane recruitment of myosin-light chain, suggesting strongly that the membrane recruitment of ROCK-1 coincides with its activation state (Fig. 4d). Moreover, fasudil that reduced ROCK-1 activity as demonstrated- by the significant reduction of P-MYPT1 and P-MLC in diabetic rats (Fig. 4b,c) induced induced a relocation of ROCK-1 and P-MLC from the membrane into the cytoplasm of GK eyes, further confirming inactivation of both proteins

**Figure 5:**
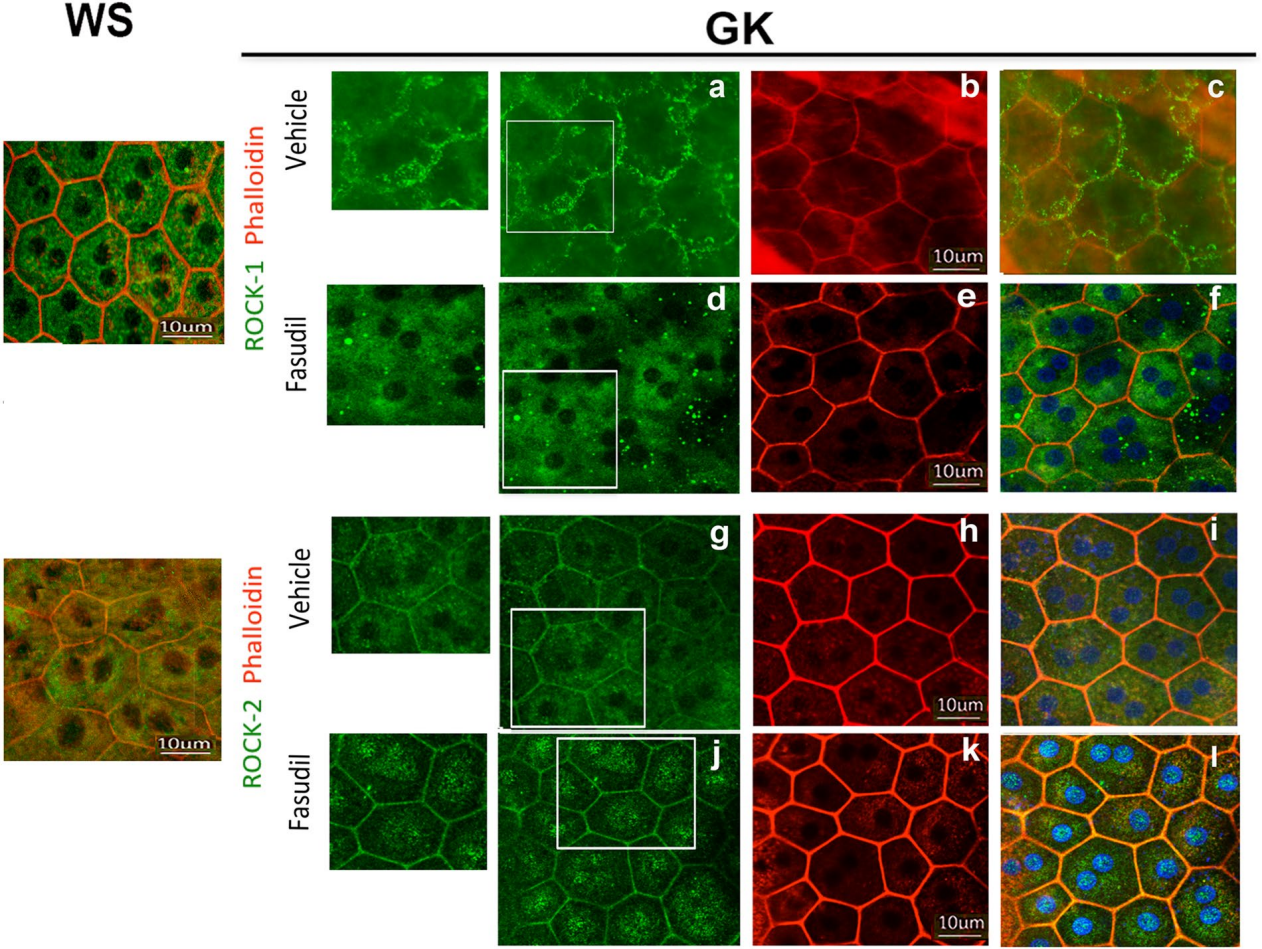
Effect of fasudil on ROCK-1 and ROCK-2 cellular distribution in diabetic RPE cells. Double staining ROCK-1 or ROCK-2 with Phalloidin on RPE cell flatmounts showed that Fasudil relocates ROCK-1 (a,b) from the membrane the cytoplasm in the Fasudil treated GK rat consistent with their inactivation and that it did not change the membrane distribution of ROCK-2 but induced an increased nuclear and perinuclear localization of ROCK-2 (g,j). (n = 5 per experiment). Scale bar = 10 µm.

Whilst fasudil slightly increased the nuclear and perinuclear distribution of ROCK-2 in RPE cells, it did not influence it membrane recruitment (Fig. 5g,j and inset magnification) and was not associated with membrane blebs. In contrast, fasudil translocated ROCK-1 back from membrane blebs (Fig. 5a and c) to the cytoplasma (Fig. 5d and f).

### Fasudil restores RPE structure and barrier function

**Figure 6:**
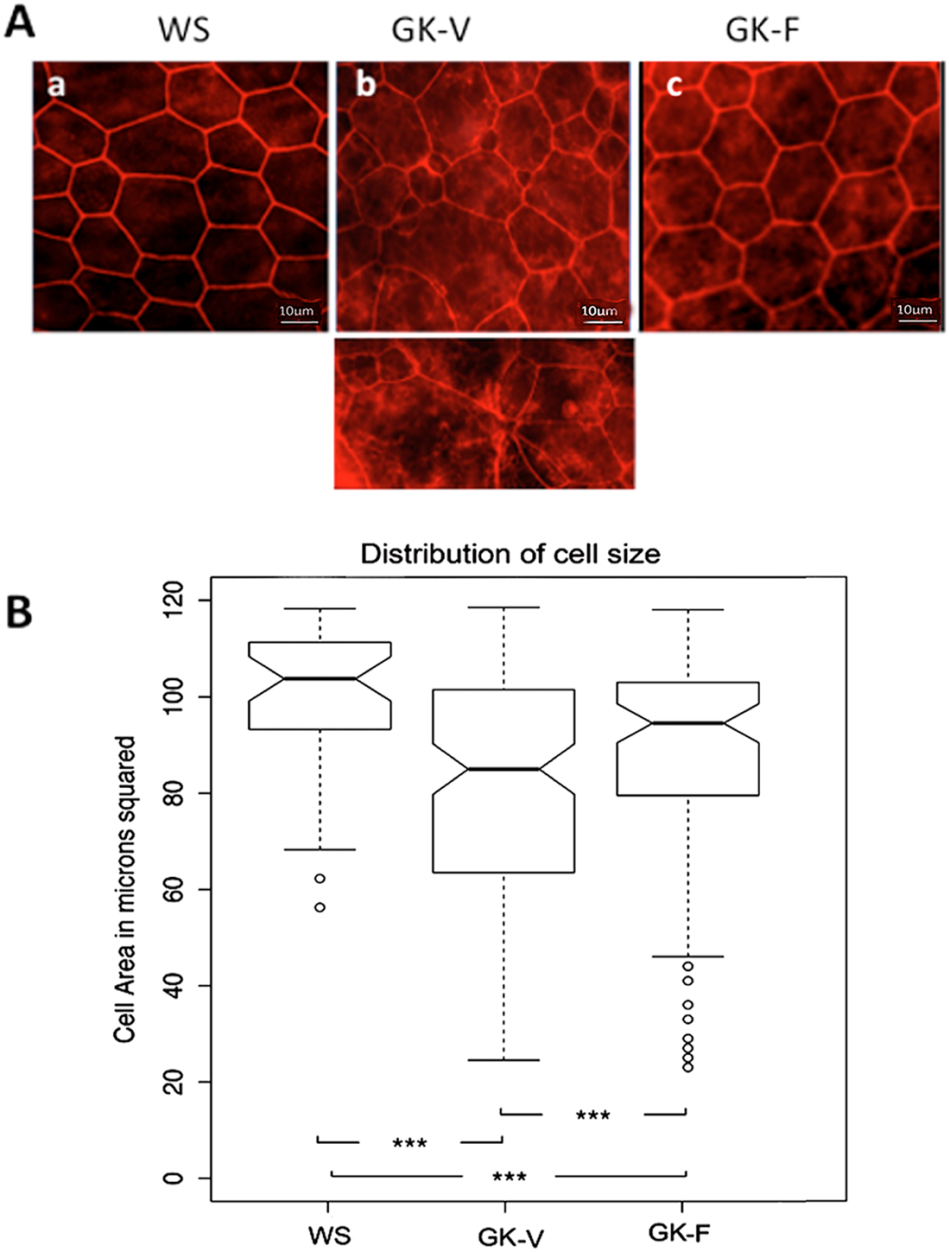
Effect of intravitreal Fasudil on RPE cell layer morphology. (**A**) Phalloidin-stained actin on flat-mounted RPE from GK rat treated *in vivo* with vehicle (GK-V) or with fasudil (GK-F). Polydispersed size of RPE with apical constriction and stress fibers in GK-V rats (b) is reverved in GK-F rats (c). Scale bar = 10 µm. (**B**) boxplot summarizing the distribution of small cells in control rats (WS) and in diabetic rats (GK) treated by Fasudil (F) or vehicle (V). The RPE of GK Fasudil treated rats had significantly less small cells than the RPE of GK vehicle treated rats, suggesting less cellular constriction (n = 7 per experiment). ***p value < 0.001.

**Figure 7:**
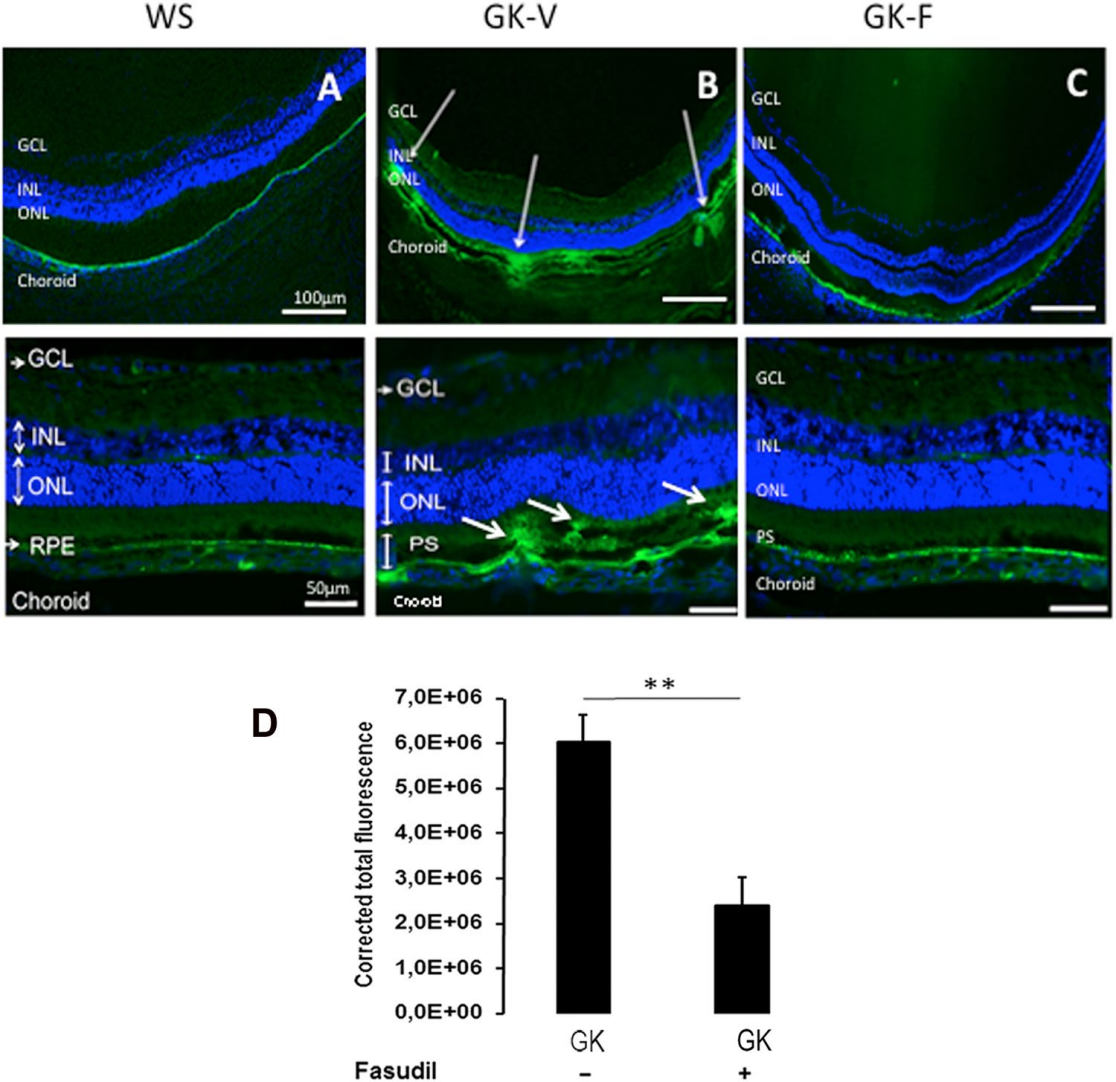
Effect of intravitreal fasudil on GK rat outer retinal barrier permeability. Two hours after an intravenous injection of 150 KDa FITC-Dextran, WS rats and GK rats treated either with vehicle (GK-V) or with fasudil (GK-F) were sacrificed and the eyes sectioned. (**A**) In WS rats, FITC-dextran remains in the choroid vessels and do not cross the RPE. (**B**) In GK rats injected with vehicle (GK-V), similar to control GK rats (not show), FITC-Dextran cross the RPE and diffuses in the outer retina and in photoreceptor segments PS (arrows). (**C**) Fasudil treatment of GK rats (GK-F) restores the RPE barrier as shown by resolution of FITC-dextran leakage in the outer retina. (**D**) Quantification of total retinal leakage was performed by the measurement of neural retina corrected total fluorescence (CTF) (see materials and methods). A significant decrease in CTF consistent with decreased leakage was found for Fasudil treated GK rats (n = 5 per experiment); **p value < 0, 01; Scale bar = 10 µm INL = Inner Nuclear Layer; GCL = Ganglion Cell Layer, ONL = Outer retinal Layer, PS = Photoreceptor Segments.

In GK rats, higher degree of RPE shape polydispersity was observed with a significant increase of very large (>400 μm^2^) and of small cells (<200 μm^2^) (Fig. 6A,b and B). Fasudil treatment significantly reduced the frequency of small cells and increased the frequency of normal size cells as compared to the GK rats treated with vehicle (Fig. 6A,c p < 0.001 and B). Fasudil treatment was thus efficient to restore the RPE monolayer morphology in GK rats. Interestingly, not only the morphology but also the function of the outer retinal barrier was restored as assessed by retinal penetration of FITC-dextran after intravenous injections (Fig. 7). Indeed, FITC dextran that remained in the choroid of WS rats and did not diffuse in the retina due to functional barrier (Fig. 7A), passively diffused from the choroid through the RPE barrier into the outer retina in diabetic rats (Fig. 7B) demonstrating a breakdown of the outer retinal barrier. In GK rats treated with fasudil, a significant reduction of FITC dextran passage into the outer retina was measured, demonstrating restoration of the barrier function (Fig. 7C and D).

### Fasudil reduces vasoconstriction and reduces retinal hypoxia of the diabetic retina

**Figure 8:**
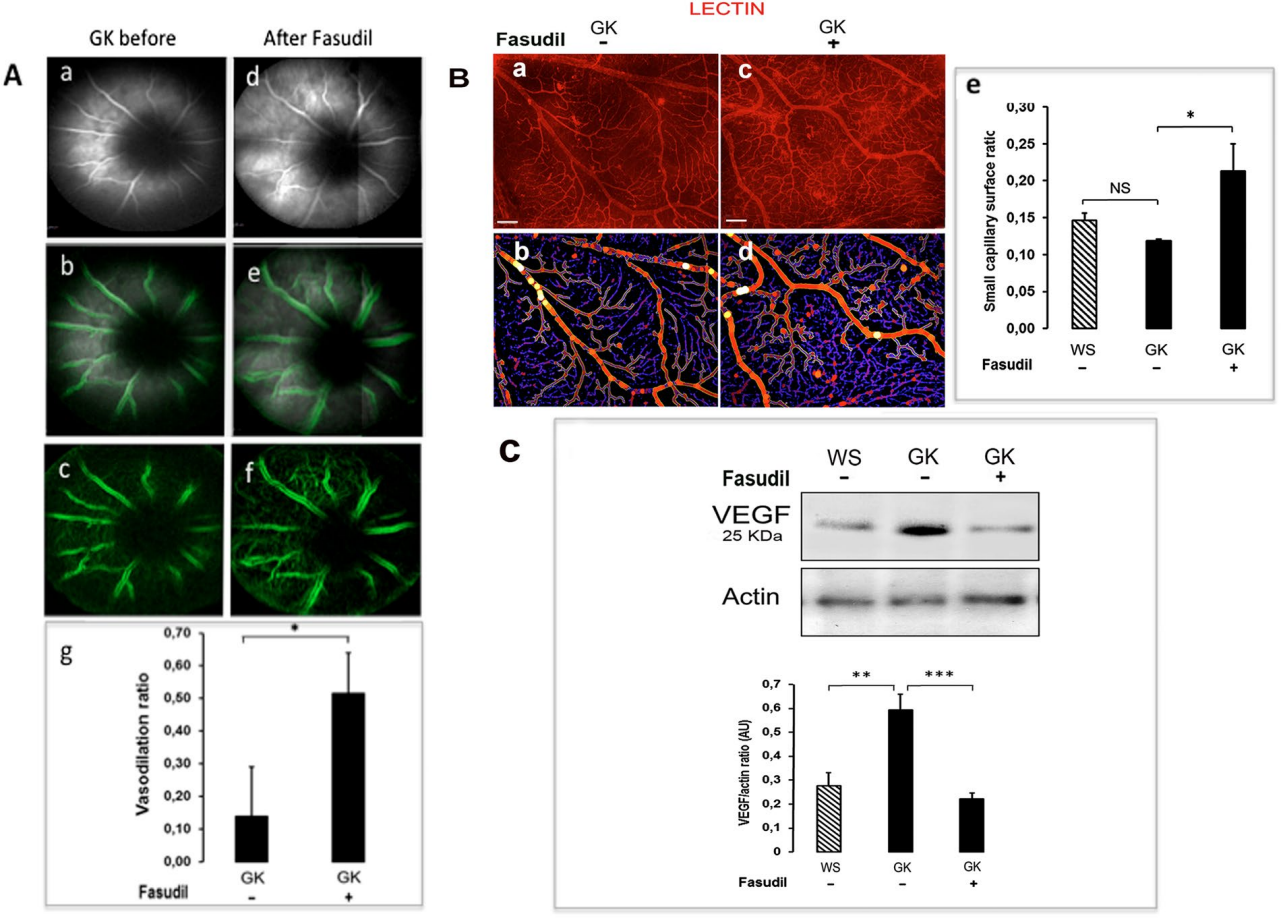
Effect of intravitreal fasudil on GK rat retinal vessel dilation. (**A**) Confocal *in vivo* fluorescein angiography before and after fasudil treatment allowed visualization of retinal vessels (a–d). Edge detection was performed using a dedicated macro (b–e,c–f) and vascular diameter before and after treatment determined a vasodilation ratio (g) showing significant effect of fasudil on retinal vessel diameter (p < 0.05). (**B**) An automated detection of retinal capillary network surface was performed on retinal flat-mounts stained by TRITC-lectin (red) to highlight the vessel walls in vehicle and Fasudil treated GK rats (small vessels represented in blue and large vessels in red) (a–b and c–d). The retinal surface coverage by small vessels was significantly increased in fasudil treated GK rats as compared to vehicle treated GK rats (n = 4–5 per group, p < 0.05) consistent with a vasodilation of retinal capillaries (e). (**C)** Western blot showed a significant decrease in the VEGF-164 level in the neural retina of fasudil treated GK rats as compared to vehicle treated GK rats, suggesting decreased retinal hypoxia in fasudil treated retina (n = 8 per group). *p value < 0, 05; **p value < 0, 01 ***p value < 0,001.

*In vivo* imaging of retinal vessels using confocal angiography showed that fasudil treatment induced a significant vasodilation of retinal vessels (Fig. 8A). Furthermore, the surface of capillary coverage, assessed on retina flat-mounts stained with lectin was significantly increased in GK-fasudil treated eyes as compared to vehicle treated eyes (Fig. 8B). Consistently, dilation of the capillary bed was associated with a significant decrease in VEGF levels (Fig. 8C) suggesting improved retinal perfusion and potentially reduced retinal ischemia.

**Figure 9:**
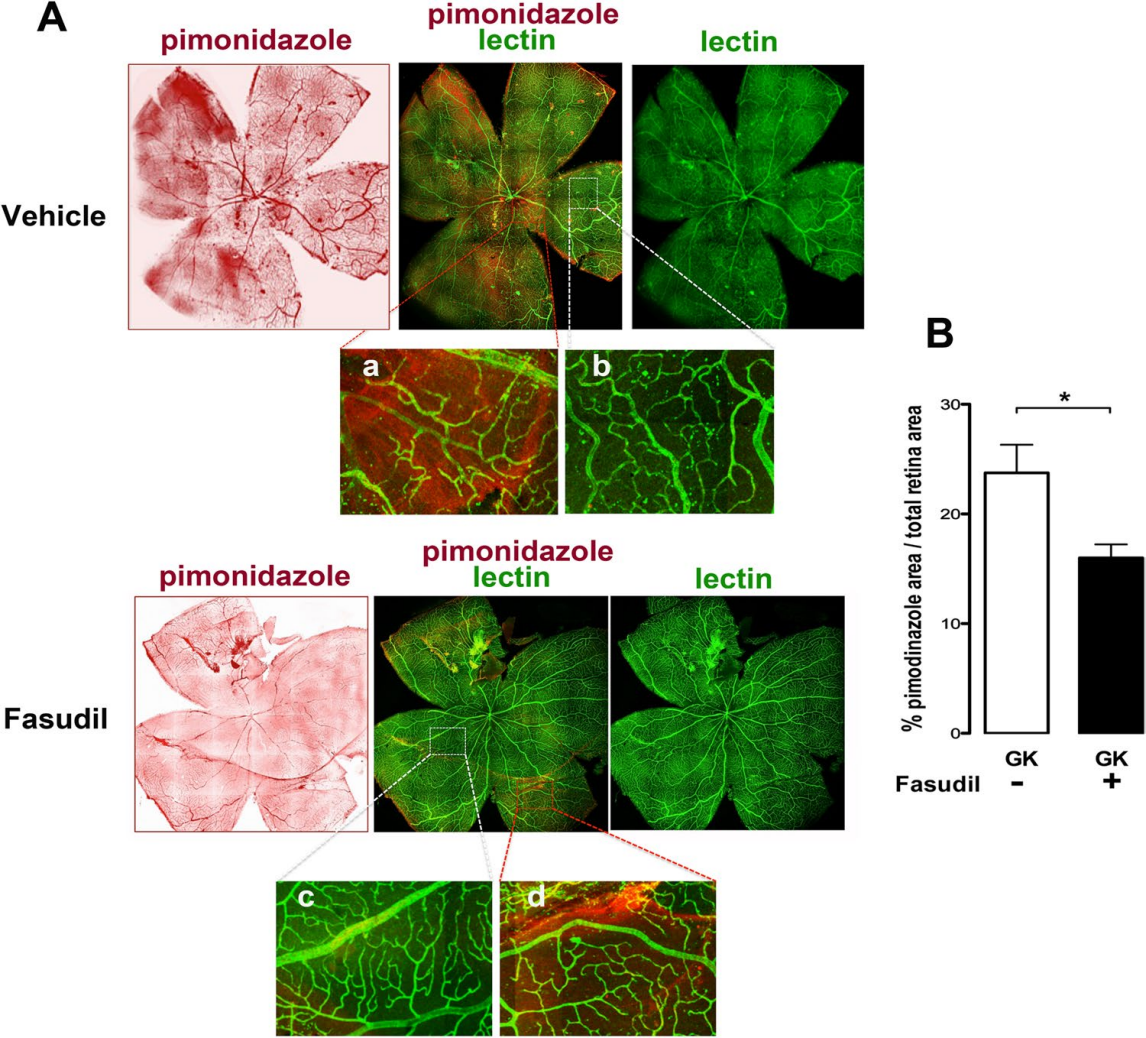
Effect of fasudil on retinal hypoxia. (**A**) Pimonidazole and lectin staining on flat-mounted retina from vehicle and fasudil treated rats. In hypoxic area stained with pimonidazole in the vehicle treated rats, lectin-labeled vessels appear constricted and irregular (a,b). Reduced hypoxic area observed in fasudil treated rats correspond to improved capillaries morphology (c) Magnification ×6. (**B**) Quantification of hypoxic aea shows that fasudil induces a significant reduction of hypoxic retinal surface ratio.

Moreover, retinal hypoxia area, which is a consequence of reduced perfusion was significantly reduced (by 33%) in GK rats treated with fasudil. Indeed, whilst in GK control rats, 24% of the retina was positively labeled with pimonidazole, only 16% of the retina was hypoxic in the fasudil treated GK rats (Fig. 9A and B). In vehicle treated GK rats, pimonidazole positively stained area coincides with low retinal capillaries coverage (Fig. 9A,a,b). In fasudil-treated rats, the improved capillaries coverage is associated with reduced pimonidazole staining (Fig. 9A,c,d).

## Dicussion

We have evidenced the activation of ROCK-1 in the retinal pigment epithelium and in retinal vessels from GK rat, based on its cellular distribution and on increased phosphorylation of its substrates, MYPT1 and MLC. Similarly, membrane localization of ROCK-1 has been observed in human retina from diabetic but not from non-diabetic individual suggesting that ROCK-1 is also activated in the RPE and in the vascular endothelium of the retina in human diabetic condition. In our experimental model, ROCK-2 is expressed in RPE and endothelial cells (not shown) but its cellular distribution is not modified by diabetes suggesting that ROCK-2 may be submitted to other diabetic-induced changes, not analyzed in this paper. The two Rho kinases functions may vary in different cell types and experimental conditions. In example, in STZ rats, the level of ROCK-1 and ROCK-2 protein expression was shown to be increased in corpus cavernosum^38^ while in GK rat retina, the ROCK-1 protein level remains stable and only its activity is enhanced. The activation of ROCK-1 in vessels under hyperglycemia has been previously shown to contribute to endothelial dysfunction^39, 40^. In the GK rat, activation of the Rho kinase pathway was suspected to contribute to hypertension and to mesenteric arteries reactivity^41^. In STZ-induced diabetes model, activation of ROCK pathway in the retinal microvasculature has been observed and rather suspected to contribute to endothelial cell death through Fas-FasL and nitric-oxide-mediated mechanisms^24^. In our model, using intravenous injection of propidium iodide we did not evidence endothelial cell death, ROCK-1 cleavage by caspase 3 which is involved in apoptotic blebbing was not observed^42^, and we could not detect the truncated form of ROCK-1 by western blotting (results not shown). Such discrepancy can be explained by the fact that streptozotocin *per se* can induce direct pro-apoptotic effects though nitric-oxide and activation of NF-□B pathways^43^ suggesting that endothelial cell death in this model may not result only from hyperglycemia but also from STZ toxicity. It might be also possible that endothelial cell death takes place at later time points in GK type 2 diabetic rats.

Our observations show that in one-year old GK rat, activation of ROCK-1 contributes to vascular closure by two mechanisms: -focal vessel wall constriction and, - formation of endothelial cell membrane blebs which narrow the capillary lumen. Such non-apoptotic cell membrane blebbing has been shown to occur in endothelial cells under oxidative stress *in vitro* ^31, 44^ and was reversed *in vivo* by anti-oxydant in cardiac capillaries after ischemia^32^. Interestingly, circulating microparticles, which result from detached blebs, carry pro coagulant, pro angiogenic and pro inflammatory messages in diabetes^33^ and can also contribute to capillary occlusion.

To date, increased leukocyte-endothelial cell adhesion/entrapment in retinal capillaries^24^, endothelial dysfunction, oxidative stress^34^, and vascular inflammation are recognized as contributing factors to capillary occlusion^45^. The results of this article indicate that through ROCK-1 activation, formation of membrane cell blebs could be another factor contributing to retinal vessels occlusion and subsequent ischemia. Fasudil injected into the vitreous exerted vasodilation on retinal vessels, and reduced hypoxic area and VEGF expression demonstrating beneficial effects of retinal ischemia. Anti-angiogenic effects associated with reduced VEGF is a known effect of fasudil^46, 47^ and in endothelial cells, fasudil inhibited hypoxia-induced HIF-1α expression and disrupted VEGF/VEGFR-2 autocrine loop^42^. Thus, as opposed to anti-VEGF that may have vasoconstrictor effects, inhibition of ROCK-1 reduces VEGF and capillary constriction together with reduced retinal hypoxia, which may be of the utmost importance in ischemic macular edema^48^. Contrarily, to irreversible vascular closure that follows endothelial cell death, the blebbing-induced closure could be reversed by ROCK inhibitors, opening a window for intervention in case of macular ischemia.

Outer retinal RPE barrier disruption in GK rats, like in diabetic patients precedes the microangiopathy^2^. Indeed, clinical studies have demonstrated a correlation between glycated hemoglobin (HbA1C), which reflects the blood glucose levels of the 3 preceding months, and increased retinal thickness, prior to the onset of diabetic retinopathy diagnosed on microangiopathy signs^49^, suggestive of RPE dysfunction in pre-clinical DR or DMO.

In the present study, we showed that in GK rat and human diabetic RPE, ROCK-1 membrane localization was associated with marked alterations of the RPE actin cytoskeleton, shape modifications (apical constriction), cell-cell junction disruption and blebbing. Apical constriction of the actin-myosin network together with stress fibers induced rupture of cell junctions resulting in functional alteration of the outer retina as demonstrated by FITC-dextran passage from the choroid through the RPE into the outer retina. Fasudil significantly reduced the retinal dextran leakage demonstrating the impact of ROCK pathway activation in RPE barrier breakdown. In response to chemical oxidative stress *in vitro* RPE membrane blebbing was previously described^49^ and RhoA/ROCK-1/P-MLC pathway activation in response to Plasminogen Activator Inhibitor 1 (PAI-1) intervene in bleb formation of human colon cancer cells^50^. PAI-1 polymorphism was recently associated with an increased risk of diabetic retinopathy^51^. Whether PAI-I intervenes in RPE blebs should be examined.

Fasudil (Asahi Kasei, Corp Tokyo, licensed to Schering) is a non-specific ROCK inhibitor approved for use in humans (only in Japan) for cerebral vasospasm after subarachnoid hemorrhage^52^. The potential benefits of ROCK inhibitors have been advocated in pre-clinical and clinical studies for the treatment of arteriosclerosis, hypertension, pulmonary hypertension, stroke, ischemia-reperfusion injury and heart failure, and in diabetic nephropathy But side-effects (such as convulsion, hypotension and disturbance in consciousness) due to the lack of specificity of available inhibitors, have so far restricted their clinical development^53–56^. More specific ROCK inhibitors are developed for improved therapeutic window. Intraocular administration of fasudil reduces the risk of systemic side-effects since the eye is a confined organ with limited systemic diffusion of intraocular injected compounds. Recently, instilled Ripasudil, another ROCK inhibitor, efficiently reduced retinal angiogenesis^56^. Because fasudil is not a specific ROCK inhibitor, we cannot ascertain that only ROCK-1 inhibition is mediating the observed beneficial effects of fasudil on the GK rat retinopathy. However, diabetic conditions did not modify the membrane distribution of ROCK-2 and ROCK-2 was not recruited in membrane blebs in GK rats. On the other hand, under diabetic conditions, ROCK-1 was clearly recruited in cell membranes and in blebs in RPE and endothelial cells of GK rats and of diabetic human retina and this recruitment was inhibited by fasudil.

The subcellular localization of ROCK-1 and ROCK-2 is dependent on the cell type. ROCK-2 localization and its association with centrosome has already been reported^57^, suggesting that the different kinases may exert differential specific functions. On the other hand, membrane blebbing was previously shown to be ROCK-1 activation dependent during apoptosis^58^. Moreover, in non-apoptotic conditions *in vitro*, cell blebbing was shown to be associated with ROCK-1 recruitment as demonstrated by GFP-tagged-ROCK-1^37^. The contribution of ROCK-2 to fasudil effect on membrane blebbing is thus uncertain since its membrane localization was not modified by fasudil treatment. This does not exclude that fasudil could have other inhibiting effects through ROCK-2 inhibition in the retina during diabetes.

In summary, this study demonstrates that in type 2 diabetic retinas, ROCK-1 activation in retinal endothelial cells and in RPE cells, induces cytoskeleton remodeling and membrane blebs, that contribute to micro-vascular closure with subsequent retinal hypoxia and to disrupted RPE barrier with leakage. Local ROCK inhibition, using intraocular injection of fasudil efficiently reversed these effects. Since fasudil is not a specific ROCK inhibitor, both ROCK-1 and ROCK-2 could be involved in fasudil effects observed *in vivo* on the diabetic retina. Further experiments using specific ROCK inhibitors, when available, could better discriminate which kinase is involved in this process. Our results, together with previous observations suggest that in diabetic retinopathy associating macular edema combined with ischemia, intraocular fasudil could reduce edema and favor retinal perfusion, opening interesting new therapeutic potential.
